# Perceived stress and depression in Chinese university students: a parallel mediation analysis examining the roles of insomnia and somatic symptoms

**DOI:** 10.3389/fpsyg.2026.1847914

**Published:** 2026-05-19

**Authors:** Jie Ren, Nana Xiong, Qi Liu, Hongyu Su, Marcus A. Rodriguez, Yusi Wang, Tengteng Fan

**Affiliations:** 1 Beijing Xicheng District Pingan Hospital, Beijing, China; 2 NHC Key Laboratory of Mental Health, Institute of Mental Health, Peking University Sixth Hospital, National Clinical Research Center for Mental Disorders, Peking University, Beijing, China; 3 The Third Hospital of Chaoyang District, Beijing, China; 4 Pitzer College, Claremont, CA, United States; 5 Loyola University Maryland, Baltimore, MD, United States

**Keywords:** depression, insomnia, mediating effect, perceived stress, somatic symptoms, university students

## Abstract

**Background:**

Previous studies have demonstrated the association between perceived stress and depression. A key question remains unclear whether insomnia and somatic symptoms function as simple manifestations of depression or serve as mediating roles in the stress-depression relationship, particularly among university students. This study explored how insomnia and somatic symptoms mediate the relationship between perceived stress and depression among university students in China.

**Methods:**

A total of 2,596 Chinese university students (68.3% female, mean age 22.3 ± 4.3) were included. The Perceived Stress Scale-10 (PSS-10), Insomnia Severity Index (ISI), Somatic Symptom Scale-8 (SSS-8), and Patient Health Questionnaire-9 (PHQ-9) were used to assess levels of perceived stress, insomnia, somatic symptoms and depression, respectively.

**Results:**

Depression screening was positive in 48.2% of participants (PHQ-9 ≥ 10), with 45.5% meeting clinical insomnia criteria (ISI ≥ 9) and 42.0% reporting significant somatic symptoms (SSS-8 ≥ 9). Perceived stress was significantly correlated with insomnia, somatic symptoms, and depression (*r* = 0.493–0.702, all *p* < 0.001). Parallel mediation analysis revealed that perceived stress exerted both a direct effect [effect size = 0.382, 95% CI (0.357, 0.407)], and indirect effects [effect size = 0.235, 95% CI (0.214, 0.254)] on depression. The indirect effects, accounting for 38.1% of the total effect, were comparably mediated through insomnia (17.3%) and somatic symptoms (20.8%).

**Conclusion:**

In this cross-sectional study, insomnia and somatic symptoms served as significant parallel mediators in the association between perceived stress and depression among university students. These findings support the potential value of including interventions targeting sleep problems and somatic symptoms in depression prevention efforts for university populations.

## 1 Introduction

Mental health challenges among university students have become a growing global public health concern, with depression being particularly prevalent, affecting approximately 27.2% of this population (Auerbach et al., 2016; Rotenstein et al., 2016). University students, at a critical developmental stage of early adulthood, face multiple challenges including academic demands, interpersonal pressures, financial concerns, and future career uncertainties (Chai and Shek, 2024; Chowdhury et al., 2022; Zhang and Zhao, 2023).

Perceived stress represents the degree to which individuals appraise situations in their lives as stressful, unpredictable, uncontrollable, and overwhelming relative to their adaptive capacity (Lazarus and Folkman, 1984). Therefore, this psychological construct reflects not merely the presence of environmental stressors, but rather how individuals evaluate both the significance of these stressors and their ability to cope with them. Although research has consistently demonstrated the association between perceived stress and an increased risk of depression (Ebert et al., 2019; Haehner et al., 2024; LeMoult et al., 2023; Zuo et al., 2020), the mechanisms underlying this relationship remain incompletely understood given to heterogeneous responses to stress among individuals. A key question remains unclear whether insomnia and somatic symptoms function as simple manifestations of depression or serve as mediating factors in the stress-depression relationship, particularly within the university students’ population.

According to the diathesis-stress model proposed by Spielman et al. (1987), stressors exceeding an individual’s vulnerability threshold can trigger insomnia. Once initiated, insomnia may become self-perpetuating through maladaptive behaviors and dysfunctional beliefs about sleep, ultimately contributing to the development of depression (Blake et al., 2018). This theoretical framework is supported by a meta-analysis revealing moderate correlations between stress and insomnia among undergraduate students (Gardani et al., 2022). Notably, recent research has shifted from viewing insomnia as merely a symptom of depression to recognizing it as an independent risk factor that may contribute to its onset (Baglioni and Riemann, 2012). Several studies have identified insomnia as a significant mediator in the stress-depression relationship (Liu et al., 2021; Mu et al., 2023). For instance, insomnia was found to mediate the effect of perceived stress on depression among the general population during the first wave of the COVID-19 pandemic in China (Mu et al., 2023), with similar findings observed among medical students during the pandemic (Liu et al., 2021). However, these studies are limited by their focus on either medical students or specific pandemic contexts. Furthermore, previous research has largely treated insomnia as a single mediator, neglecting its potential concurrent effects with other stress-related symptoms. Therefore, it remains unclear in understanding how multiple mediating pathways, particularly the interaction between insomnia and somatic symptoms, influence the stress-depression relationship in the broader university student population.

Like insomnia, somatic symptoms have been recognized as common manifestations of depression (Li et al., 2023; Novick et al., 2015), as well as common stress-related outcomes. Research has shown that elevated stress perception is associated with various somatic discomforts, including palpitations, shortness of breath, gastrointestinal discomforts, pain, and fatigue (Burton et al., 2020; Creed, 2022; Petersen et al., 2024). Although the etiology of chronic somatic symptoms is multifactorial, substantial evidence has demonstrated significant associations between adverse life events, perceived stress and somatic symptoms in general populations (Petersen et al., 2023; Petersen et al., 2024; Weinreich Petersen et al., 2022). In addition, the association between somatic symptoms and depression has been consistently observed across various cultural contexts (Hamamura and Mearns, 2019; Van Houdenhove et al., 2010), with longitudinal population-based cohort studies suggesting a potential causal linking (Dijkstra-Kersten et al., 2017; Dijkstra-Kersten et al., 2015; Jiang et al., 2024). However, whether somatic symptoms serve as a mediator in the stress-depression relationship, particularly in conjunction with insomnia among university students, remains inadequately explored.

In summary, despite these established associations and potential underlying biological mechanisms, three critical gaps exist in current research: (1) most studies have examined insomnia or somatic symptoms in isolation, neglecting their potential concurrent effects; (2) the specific mechanisms through which these factors interact to influence depression development remain unclear; and (3) the generalizability of existing findings to the broader university student population remains uncertain. Therefore, to address these gaps, the current study aimed to develop a parallel mediation model examining how both insomnia and somatic symptoms mediate the relationship between perceived stress and depression among Chinese university students. The potential parallel mediating effects of insomnia and somatic symptoms in the stress-depression relationship are supported by neurobiological evidence (LeMoult et al., 2023). Specifically, chronic stress may contribute to depression through distinct but related pathways: disruption of sleep–wake regulation leading to insomnia (Gold, 2015), and activation of low-grade inflammation responses manifesting as somatic symptoms (Demitrack and Crofford, 1998; Haack et al., 2007). Our findings could inform the development of more targeted and effective intervention strategies for improving student mental health.

## 2 Materials and methods

### 2.1 Participants and procedures

This cross-sectional survey was embedded within a larger project designed to enhance university students’ mental health through online dialectical behavior therapy (DBT) skills training and peer support interventions. Between May 12, 2023, and May 7, 2024, participants were recruited nationwide through a specifically developed WeChat mini-program titled “DBT Skills Learning Platform.” Recruitment was facilitated through university faculty members and students across China using convenience sampling.

This study invited eligible undergraduate and postgraduate students to participate in the free mental health assessment program. After completing the assessment, participants received individualized assessment feedback and customized health education materials. Those identified with moderate to severe levels of emotional burdens would be offered opportunities for subsequent interventions.

The assessment required approximately 15 min to complete. Before participation, all students were required to read and provide electronic informed consent. To ensure data quality, the WeChat mini-program was designed with built-in quality control measures that prompted participants to complete all unanswered items. This study was approved by the Ethics Committee of Peking University Sixth Hospital (Approval No.: 2023–28).

### 2.2 Measures

While the overall project employed a comprehensive assessment battery to assess depression, anxiety, perceived stress, insomnia, somatic symptoms, and other common mental health and behavioral problems among university students, this study focused on the following measures:

#### 2.2.1 Socio-demographic characteristics

The questionnaire collected socio-demographic information including gender, age, residential location, ethnicity, educational level, living situation, and the family economic status. Health-related data were also gathered, including physical activity patterns, smoking status, and alcohol consumption. Participants’ psychiatric history was assessed through two items: any previous psychiatric diagnoses, and any history of mental health treatment.

#### 2.2.2 Perceived Stress Scale-10 (PSS-10)

The PSS-10 is a widely used self-report scale to assess psychological stress over the past month (Cohen et al., 1983). This study utilized the 10-item Chinese version, which has demonstrated satisfactory psychometric properties among university students (Lu et al., 2017). Items are scored on a 5-point Likert scale ranging from 0 (“Never”) to 4 (“Very often”). Items 4, 5, 7, and 8 are reverse scored. Total scores range from 0 to 40, with higher total scores indicating greater perceived stress. The scale demonstrated good internal consistency in this study (Cronbach’s *α* = 0.824).

#### 2.2.3 Insomnia Severity Index (ISI)

The ISI is a self-report scale assessing the severity of insomnia over the past 2 weeks through seven items. Total scores range from 0 to 28, with higher scores indicating more severe insomnia (Bastien et al., 2001). The Chinese version has demonstrated good psychometric properties (Lin et al., 2018), with an optimal cut-off score of 9 (Chung et al., 2011). Internal consistency in this study was excellent (Cronbach’s *α* = 0.895).

#### 2.2.4 Somatic Symptom Scale-8 (SSS-8)

The SSS-8 is a reliable and valid self-report scale for assessing the burden of somatic symptoms (Gierk et al., 2014). It assesses the burden of various common somatic symptoms including gastrointestinal, pain, fatigue, and cardiopulmonary discomforts over the past 2 weeks. The eight items are scored on a 5-point Likert scale ranging from 0 (“Not bothered at all”) to 4 (“Extremely bothered”), yielding total scores of 0–32. Higher scores indicate more severe somatic symptoms. The Chinese version has a validated cutoff score of 9 (Li et al., 2022). The scale showed good internal consistency with a Cronbach’s *α* of 0.850 in this study.

#### 2.2.5 Patient Health Questionnaire-9 (PHQ-9)

The PHQ-9 is a self-report scale evaluating depressive symptoms over the past 2 weeks, based on the diagnostic criteria for depressive episodes in Diagnostic and Statistical Manual of Mental Disorders, Fourth Edition (DSM-IV). It was proved to be reliable and valid among Chinese university students (Du et al., 2017). Higher scores indicate more severe depression, with a cut-off score of 10 optimally detecting major depression (He et al., 2020). The internal consistency was high with a Cronbach’s *α* of 0.887.

### 2.3 Statistical analysis

For descriptive data, continuous variables were presented as mean and standard deviations (M ± SD), and categorical variables as frequencies and percentages. Pearson correlation coefficients were computed to examine the bivariate relationships among perceived stress, insomnia, somatic symptoms and depression. To test the hypothesized parallel mediation effects, we employed the Hayes’ PROCESS macro (Model 4) for SPSS. Direct and indirect effects was tested using bootstrapping procedures with 5,000 resamples. Demographic variables of gender, age, educational level, and history of psychiatric disorders were introduced in the model as covariates, based on their established or potential associations with the main study variables (perceived stress, insomnia, somatic symptoms, and depression) in prior literature (Fond et al., 2019; Gao et al., 2020; Liu et al., 2021). Other socio-demographic and lifestyle variables (e.g., living situation, family income, physical activity, smoking, and alcohol use) were not included as covariates in the final model to maintain model parsimony. Mediation effects were considered significant if the 95% confidence interval (CIs) excluded zero. All statistical tests were two-tailed, with statistical significance set at *p* < 0.05. All analyses were performed using IBM SPSS version 27.0 (IBM Corp. Armonk, NY, USA).

## 3 Results

### 3.1 Socio-demographic and lifestyle characteristics of Chinese university students

A total of 2,596 university students participated in the study, of whom 68.3% (*n* = 1,773) were female, with a mean age of 22.3 years (SD = 4.3) (see Table 1). The sample comprised 62.3% (*n* = 1,618) undergraduate students and 37.7% (*n* = 978) postgraduate students (including both master’s and doctoral candidates). Participants were recruited from all major geographic regions of China, with the largest proportion from North China (49.6%, *n* = 1,288). 75.1% (*n* = 1,949) participants resided in university dormitories, and the majority reported a monthly household income of 4,000–8,000 RMB (43.0%, *n* = 1,116).

**Table 1:** Socio-demographic characteristics of study participants (*n* = 2,596).

Variables	Groups	*n*	%
Gender	Male	823	31.7%
	Female	1773	68.3%
Residential location of China	Northeast China	241	9.3%
	North China	1,288	49.6%
	East China	358	13.8%
	Central China	259	10.0%
	Northwest China	133	5.1%
	Southwest China	118	4.5%
	South China	199	7.7%
Ethnicity	Han Chinese	2,300	88.6%
	Others	296	11.4%
Educational level	Undergraduate	1,618	62.3%
	Postgraduate	978	37.7%
Living situation	On-campus residence	1949	75.1%
	Off-campus rental	381	14.7%
	Living with family	266	10.2%
Per capita monthly household income (RMB, ¥)	<4,000	747	28.8%
	4,000–8,000	1,116	43.0%
	>8,000	733	28.2%
Physical exercise (summer, weekly)	>2 h	581	22.4%
	1–2 h	583	22.5%
	<1 h	863	33.2%
	No exercise	569	21.9%
Physical exercise (winter, weekly)	>2 h	384	14.8%
	1–2 h	463	17.8%
	<1 h	953	36.7%
	No exercise	796	30.7%
Smoking status	Never smoker	2,267	87.3%
	Former smoker	167	6.4%
	Current smoker	162	6.2%
Alcohol consumption pattern	Never drinker	1,181	45.5%
	Occasional drinker	1,312	50.5%
	Former drinker	69	2.7%
	Regular drinker (>3 days/week)	34	1.3%
Any previous psychiatric diagnoses	Yes	541	20.8%
	No	2055	79.2%
Any history of mental health treatment	Yes	501	19.3%
	No	2095	80.7%

Regarding lifestyle behaviors, physical activity levels varied seasonally, with fewer students engaging in regular exercise during winter (>2 h/week: 14.8%) compared to summer (>2 h/week: 22.4%). The proportion of physically inactive students (no exercise) increased from 21.9% in summer to 30.7% in winter. Most participants never smoked (87.3%) or consumed alcohol regularly, with only a small proportion reported being regular drinkers (1.3%). Notably, approximately one-fifth of participants (20.8%, *n* = 541) reported having been diagnosed with certain psychiatric disorders, and most of these individuals (19.3%, *n* = 501) had received mental health treatment accordingly.

### 3.2 Prevalence and correlations of psychosomatic symptoms in Chinese university students

The severity of psychological and somatic symptoms is presented in Table 2. The mean score of perceived stress (PSS-10) was 18.8 (SD = 6.3). Based on validated cut-off scores, 45.5% (*n* = 1,181) met the criteria for clinical insomnia (ISI ≥ 9) and 42.0% (*n* = 1,090) reported significant somatic symptoms (SSS-8 ≥ 9), respectively. Depression screening was positive in 48.2% (*n* = 1,250) of participants (PHQ-9 ≥ 10). Gender comparison revealed that female students reported significantly higher levels of perceived stress (*p* = 0.019) and somatic symptoms (*p* < 0.001) than male students. Analysis by educational level showed that undergraduate students scored significantly higher on all psychosomatic measures compared to postgraduate students (all *p* < 0.01). Pearson correlation analyses demonstrated significant positive associations between perceived stress and other symptoms (insomnia, somatic symptoms, and depression; *r* = 0.493–0.702, all *p* < 0.001). Both insomnia and somatic symptoms were strongly correlated with depression (*r* = 0.662 and *r* = 0.688, respectively, both *p* < 0.001).

**Table 2:** Mean scores of psychosomatic symptoms by gender and educational level (*n* = 2,596).

Variables	Total	Gender		Educational level	
		Male	Female	Undergraduate	Postgraduate
Perceived stress (PSS-10)	18.8 ± 6.3	18.4 ± 6.2	19.0 ± 6.4^*^	19.1 ± 6.3	18.3 ± 6.4^*^
Insomnia (ISI)	8.6 ± 5.6	8.7 ± 6.0	8.5 ± 5.5	8.9 ± 5.8	8.1 ± 5.3^*^
Somatic symptoms (SSS-8)	8.4 ± 6.0	7.6 ± 6.0	8.7 ± 5.9^*^	8.8 ± 6.2	7.6 ± 5.4^*^
Depression (PHQ-9)	10.0 ± 5.9	9.9 ± 6.1	10.0 ± 5.7	10.4 ± 6.0	9.3 ± 5.5^*^

Data are presented as mean ± standard deviation. PSS-10, Perceived Stress Scale-10; ISI, Insomnia Severity Index; SSS-8, Somatic Symptom Scale-8; PHQ-9, Patient Health Questionnaire-9. ^*^Indicates significant differences between groups (*p* < 0.05).

### 3.3 Mediating effects of insomnia and somatic symptoms

**Figure 1:**
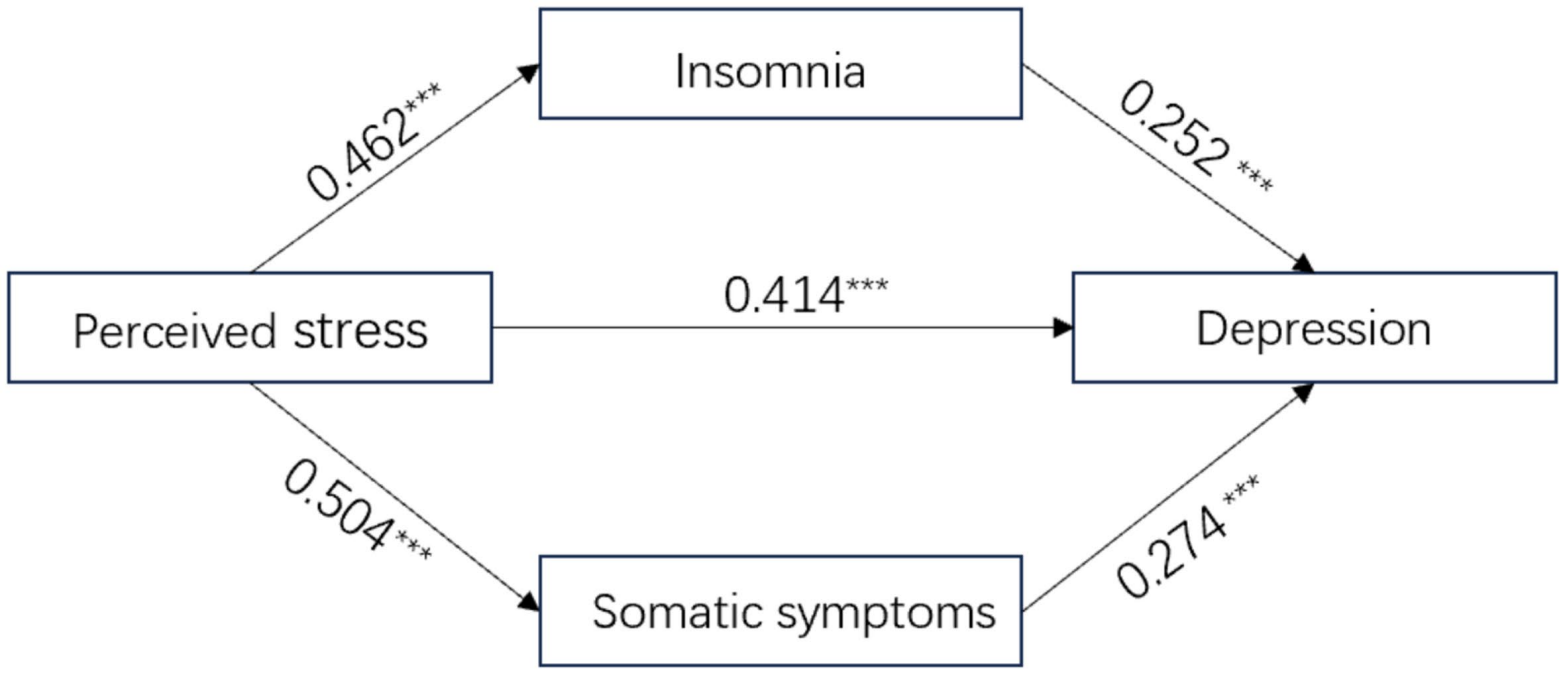
Standardized path coefficients for the parallel mediation model examining the relationship between perceived stress and depression through insomnia and somatic symptoms. The model was adjusted for gender, age, educational level, and history of psychiatric disorders. Numbers represent standardized regression coefficients (*β*). All paths depicted were statistically significant at *p* < 0.001.

Based on our hypothesis, a parallel mediation model was tested with perceived stress as the predictor, depression as the outcome, and insomnia and somatic symptoms as parallel mediators (Figure 1). Demographic variables of gender, age, educational level, and history of psychiatric disorders were included as covariates. Preliminary regression analyses were conducted to test the prerequisites for mediation. As shown in Table 3, perceived stress significantly predicted both potential mediators: insomnia (*β* = 0.462, *p* < 0.001; model 1) and somatic symptoms (*β* = 0.462, *p* < 0.001; model 2). In model 3, when all predictors were entered, perceived stress maintained a significant independent predictor on depression (*β* = 0.414, *p* < 0.001), as well as both insomnia (*β* = 0.252, *p* < 0.001) and somatic symptoms (*β* = 0.274, *p* < 0.001) significantly predicted depression. These significant associations supported testing the proposed mediation model.

**Table 3:** Regression analyses testing paths in the mediation model (*n* = 2,596).

Variables	Perceived stress → insomnia (model 1)				
	*B*	SE	*β*	*t*	*p*
Gender	−0.492	0.205	−0.041	−2.398	0.017
Age	0.015	0.028	0.011	0.533	0.594
Educational level	−0.386	0.247	−0.033	−1.560	0.119
Psychiatric history	2.202	0.239	0.159	9.222	<0.001
Perceived stress (PSS-10)	0.411	0.015	0.462	26.840	<0.001

Variables	Perceived stress → somatic symptoms (model 2)				
	*B*	SE	*β*	*t*	*p*
Gender	0.841	0.208	0.066	4.043	<0.001
Age	−0.019	0.028	−0.014	−0.678	0.498
Educational level	−0.755	0.251	−0.061	−3.013	0.003
Psychiatric history	2.201	0.242	0.150	9.088	<0.001
Perceived stress (PSS-10)	0.474	0.016	0.504	30.514	<0.001

Variables	Perceived stress, insomnia, somatic symptoms → depression (model 3)				
	*B*	SE	*β*	*t*	*p*
Gender	−0.317	0.144	−0.025	−2.202	0.028
Age	−0.014	0.019	−0.011	−0.749	0.454
Educational level	−0.209	0.172	−0.017	−1.210	0.226
Psychiatric history	1.080	0.170	0.075	6.363	<0.001
Perceived stress (PSS-10)	0.382	0.013	0.414	30.120	<0.001
Insomnia (ISI)	0.261	0.016	0.252	15.915	<0.001
Somatic symptoms (SSS-8)	0.269	0.016	0.274	16.631	<0.001

SE, Standard Error; PSS-10, Perceived Stress Scale-10; ISI, Insomnia Severity Index; SSS-8, Somatic Symptom Scale-8; PHQ-9, Patient Health Questionnaire-9.

In the mediation analysis, bootstrap analyses with 5,000 resamples confirmed significant direct and indirect effects of the hypothesized model (Figure 1 and Table 4). The total effect of perceived stress on depression was 0.617 [95% CI (0.592, 0.642)], with the direct effect accounting for 61.9% [effect size = 0.382, 95% CI (0.357, 0.407)]. The total indirect effect through both mediators was 0.235 [95% CI (0.214, 0.254)], accounting for 38.1% of the total effect. Specifically, insomnia mediated 17.3% of the total effect [effect size = 0.107, 95% CI (0.090, 0.125)], while somatic symptoms mediated 20.8% [effect size = 0.128, 95% CI (0.110, 0.146)]. A permutation test revealed no significant difference between these two indirect effects [−0.020, 95% CI (−0.049, 0.008)].

**Table 4:** Bootstrap analysis of direct and indirect effects in the parallel mediation model (*n* = 2,596).

Effect type	Effect size	SE	95%CI	Proportion
Total effect	0.617	0.013	(0.592, 0.642)	100.0%
Direct effect	0.382	0.013	(0.357, 0.407)	61.9%
Total indirect effect	0.235	0.010	(0.214, 0.254)	38.1%
Via insomnia	0.107	0.009	(0.090, 0.125)	17.3%
Via somatic symptoms	0.128	0.009	(0.110, 0.146)	20.8%
Contrast (insomnia vs. somatic symptoms^a^)	−0.020	0.015	(−0.049, 0.008)	

SE, Standard Error; CI, confidence interval. ^a^Negative values indicate that the indirect effect through somatic symptoms was larger than through insomnia.

## 4 Discussion

This study investigated the mediating roles of insomnia and somatic symptoms in the relationship between perceived stress and depression among university students. Our findings revealed significant parallel mediating pathways, suggesting potential mechanisms that may account for the cross-sectional association between perceived stress and depression in this population.

Consistent with previous research, we found significant correlations between depression and perceived stress (Fawzy and Hamed, 2017; Liu et al., 2024; Zuo et al., 2020), as well as between depression and both insomnia and somatic symptoms among university students (Hamamura and Mearns, 2019; Li et al., 2024; Marino et al., 2021). These findings prompted us to examine whether these stress-related physiological dysfunctions mediate the stress-depression relationship.

Our findings supported the diathesis-stress model, demonstrating that elevated perceived stress is linked to depression via insomnia. The mediating role of insomnia is consistent with previous studies across different age cohorts (Liu et al., 2017; Mu et al., 2023), and extends findings from pandemic contexts (Liu et al., 2021). Mechanistically, heightened perceived stress is associated with psychophysiological hyperarousal and disrupts circadian rhythm homeostasis, potentially leading to insomnia problems which could manifest as difficulties in sleep initiation, maintenance, and quality (Benham, 2019; Gardani et al., 2022; Zheng et al., 2022). Subsequently, insomnia is linked to emotional regulation (Li et al., 2024), potentially precipitating depressive symptoms.

In addition, our findings highlight the mediating role of somatic symptoms, an aspect less explored in previous research. Stress-induced somatic symptoms, such as headaches, back pain, and indigestion (Creed, 2022; Petersen et al., 2023; Petersen et al., 2024), are particularly prevalent in individuals with alexithymia and neuroticism (Zunhammer et al., 2013). The underlying mechanisms may involve stress-activated sympathetic nervous system, elevated stress hormones including cortisol (Nyengaard et al., 2024), and chronic low-grade inflammation (Koudouovoh-Tripp et al., 2021), which collectively affect physiological functioning. Recent neuroimaging evidence using resting-state functional magnetic resonance imaging (fMRI) has identified specific neural pathways underlying the mediating effect of somatic symptoms in the stress-depression relationship (Kong et al., 2022).

Notably, insomnia and somatic symptoms demonstrated comparable mediating effects in the stress-depression relationship, collectively accounting for 38.1% of the total effect. This finding emphasizes the equal importance of both physiological responses to stress. Therefore, both symptoms can serve as potential early indicators of stress responses, and their recognition may facilitate timely intervention to prevent the development of depression.

This study has some limitations. First, the cross-sectional design precludes causal inferences among the studied variables. Accordingly, the observed mediation effects should be interpreted as cross-sectional indirect associations rather than causal pathways. Longitudinal studies are needed to establish temporal relationships and confirm the directionality of these associations. Second, although perceived stress, insomnia, somatic symptoms, and depression are theoretically distinct constructs, reliance on self-report measures for these variables may introduce common method bias, potentially inflating the observed associations. Future research would benefit from incorporating clinical interviews (e.g., the Mini-International Neuropsychiatric Interview, the Structured Clinical Interview for DSM Disorders) and objective measures (e.g., polysomnography for sleep assessment) to improve diagnostic accuracy. Third, although our sample included students from various regions of China, several sampling limitations should be noted. The use of convenience sampling may affect the representativeness of our findings, and self-selection bias might have occurred, as students experiencing emotional distress may have been more motivated to participate. This potential sampling bias could explain our higher depression prevalence compared to previous epidemiological surveys. Future studies using stratified random sampling across different universities could provide more generalizable results.

## 5 Conclusion

In summary, this cross-sectional study shows that insomnia and somatic symptoms serve as significant parallel mediators in the observed association between perceived stress and depression among university students. These findings suggest that incorporating interventions targeting sleep problems and somatic symptoms into depression prevention efforts could be beneficial for university populations, though causal interpretations are not warranted by the current design.

## Data Availability

The original contributions presented in the study are included in the article/supplementary material, further inquiries can be directed to the corresponding authors.
